# Bridging Molecular Docking to Molecular Dynamics in Exploring Ligand-Protein Recognition Process: An Overview

**DOI:** 10.3389/fphar.2018.00923

**Published:** 2018-08-22

**Authors:** Veronica Salmaso, Stefano Moro

**Affiliations:** Molecular Modeling Section, Department of Pharmaceutical and Pharmacological Sciences, University of Padova, Padova, Italy

**Keywords:** ligand-protein binding, molecular docking, molecular dynamics, enhanced sampling, protein flexibility, molecular recognition

## Abstract

Computational techniques have been applied in the drug discovery pipeline since the 1980s. Given the low computational resources of the time, the first molecular modeling strategies relied on a rigid view of the ligand-target binding process. During the years, the evolution of hardware technologies has gradually allowed simulating the dynamic nature of the binding event. In this work, we present an overview of the evolution of structure-based drug discovery techniques in the study of ligand-target recognition phenomenon, going from the static molecular docking toward enhanced molecular dynamics strategies.

## Introduction

No protein is an island but exerts its function through the recognition of other molecular partners (Salmaso, 2018). Ligand-protein interactions are involved in many biological processes with consequent pharmaceutical implications. Thus, the scientific community has been putting a great effort into the investigation of the binding phenomenon during the years, leading to the proposal of several theories characterized by an increasing emphasis on the degree of flexibility of the ligand and protein counterparts.

The first explanation of binding was provided by Emil Fischer in 1894 (Fischer, 1894) with the “lock-key” model to interpret enzyme specificity: the ligand rigidly recognizes and occupies the protein binding site like a key to its lock, because of their native shape complementary. Since this model could not explain either the behavior of enzyme noncompetitive inhibition or allosteric modulation, different modifications have been proposed. Koshland (1958) introduced the “induced-fit” theory: according to his observations on enzyme-substrate interactions, the ligand is able to induce conformational changes to the protein, optimizing ligand-target interactions. Later works suggested that proteins naturally exist as an ensemble of conformations (Monod et al., 1965), described by an energy landscape (Frauenfelder et al., 1991), and ligands preferentially bind to one of them (Austin et al., 1975; Foote and Milstein, 1994). According to this interpretation of binding, known as “conformational selection,” the ligand stabilizes one of the protein conformations with a consequent shift of the protein population equilibrium (Kumar et al., 2000). These two apparently contrasting theories have simply different ranges of applicability, and the descriptions they provide of molecular binding differ for the chronological sequence of events in which the binding process is decomposed (Kobilka and Deupi, 2007; Okazaki and Takada, 2008; Zhou, 2010). New theories are emerging, making a compromise between the aforementioned ones: according to the extended conformational selection model, for example, the conformational selection is followed by a conformational adjustment (induced fit) (Csermely et al., 2010).

The evolution of binding models has practical relevance besides an epistemological significance; the knowledge of ligand-target binding is at the basis of rational drug design but understanding this complex process on a mechanistic level may open new scenarios. In addition, to suggest ligand modification meant to optimize the final bound state, the medicinal chemist may look at kinetically relevant intermediate states and try to affect them.

## Computational methods to study ligand-protein binding

Since the 1980s, computer technologies have been applied to the drug discovery process (Van Drie, 2007), giving rise to Computer-Aided Drug Design (CADD). This technique earned soon great interest and deserved a cover article on October 5, 1981, Fortune magazine, entitled “Next Industrial Revolution: Designing Drugs by Computer at Merck” (Van Drie, 2007). CADD techniques are used principally for three reasons: virtual screening *hit*/*lead* optimization and design of novel compounds. In virtual screening a huge database of compounds is examined searching for binding capacity for a target and a subset of compounds is picked out and suggested for *in vitro* testing; the purpose is to increase the *hit* rate of novel drugs by reducing the number of compounds to test experimentally. The second application of CADD is the optimization of a *hit/lead* compound driven by the rationalization of a structure-activity relationship. After the individuation of key elements for binding, the design of new compounds can be attempted (Salmaso, 2018).

CADD methods may be classified as ligand-based (LB) and structure-based (SB), depending on the availability and employment of the target structure (Sliwoski et al., 2014). In the framework of CADD, structure-based drug design (SBDD) methods take advantage of the abundance of experimentally solved structures in the Protein Data Bank (Berman et al., 2000), which can possibly be used also as templates for homology models if the structure of interest is lacking. SBDD is based on the premise that the knowledge of the target structure can help to rationalize and optimize binding since ligand-target interactions are mediated by their complementarity. With the evolution of the binding models, it is clear that speaking of “target structure” is an approximation, given that proteins fluctuate among an ensemble of structures (Miller and Dill, 1997).

The possibility to predict ligand binding modes and to interpret binding processes is valuable to individuate, optimize and suggest novel ligands, and for this reason, the scientific community has been putting great efforts in developing new computational techniques.

In the following paragraphs, we will present an excursus over the main structure-based computational techniques employed in drug discovery. An urgency to simulate protein flexibility throughout binding has been experienced over the years, arising from the evolution of the binding models from static to dynamic. The inclusion of flexibility features in conformational sampling entails an increase in the number of degrees of freedom of the system, and consequently in the computational effort. For this reason, the development of computational tools has been occurring in parallel and thanks to the continuous improvement of hardware technologies.

### Molecular docking

Molecular docking techniques aim to predict the best matching binding mode of a ligand to a macromolecular partner (here just proteins are considered). It consists in the generation of a number of possible conformations/orientations, i.e., poses, of the ligand within the protein binding site. For this reason, the availability of the three-dimensional structure of the molecular target is a necessary condition; it can be an experimentally solved structure (such as by X-ray crystallography or NMR) or a structure obtained by computational techniques (such as homology modeling) (Salmaso, 2018).

Molecular docking is composed mainly by two stages: an engine for conformations/orientations sampling and a scoring function, which associates a score to each predicted pose (Abagyan and Totrov, 2001; Kitchen et al., 2004; Huang and Zou, 2010). The sampling process should effectively search the conformational space described by the free energy landscape, where energy, in docking, is approximated by the scoring function. The scoring function should be able to associate the native bound-conformation to the global minimum of the energy hypersurface.

#### Scoring functions

Scoring functions play the role of poses selector, used to discriminate putative correct binding modes and binders from non-binders in the pool of poses generated by the sampling engine.

There are essentially three types of scoring functions:

1. Force-field based scoring functions:

Force-field is a concept typical of molecular mechanics (see Box 1) which approximates the potential energy of a system with a combination of bonded (intramolecular) and nonbonded (intermolecular) components. In molecular docking, the nonbonded components are generally taken into account, with possibly the addition of the ligand-bonded terms, especially the torsional components. Intermolecular components include the van der Waals term, described by the Lennard-Jones potential, and the electrostatic potential, described by the Coulomb function, where a distance-dependent dielectric may be introduced to mimic the solvent effect. However, additional terms have been added to the force-field scoring functions, such as solvation terms (Brooijmans and Kuntz, 2003).

Box 1 **Molecular mechanics.**

Molecular mechanics is a method which approximates the treatment of molecules with the laws of classical mechanics, in order to limit the computational cost required for quantum mechanical calculations (Vanommeslaeghe et al., 2014). Atoms are considered as charged spheres connected by springs, neglecting the presence of electrons, in accordance with Born-Oppenheimer approximation (Born and Oppenheimer, 1927). The potential energy is approximated by a simple function which is called force-field; it is the sum of bonded (intramolecular) and nonbonded energy terms. The basic form of the function comprise bond stretching and bending described by harmonic potential, and torsional potential described by a trigonometric function, in the bonded portion. Nonbonded terms consist of van der Waals and Coulomb electrostatic interactions between couples of atoms.

As an example, these basic components of the CHARMM [78] force field are reported in the following equations

$$\begin{array}{l} V=\;V_{bonded}+\;V_{nonbonded} \end{array}$$

$$\begin{array}{ll} V_{bonded}=\underset{bonds}{\sum }K_{b}{\left(b-\;b_{0}\right)}^{2}+\;\underset{angles}{\sum }K_{\theta }{\left(\theta -\;\theta_{0}\right)}^{2}\\ + & \;\underset{dihedrals}{\sum }K_{\chi }\left(1+\cos\left(n\chi -\;\delta \right)\right)\\ V_{nonbonded}=\;\underset{\begin{array}{c} nonbonded\;\\ pairs\;ij \end{array}}{\sum }\frac{q_{i}q_{j}}{\varepsilon r_{ij}}\\ + & \;\underset{\begin{array}{c} nonbonded\;\\ pairs\;ij \end{array}}{\sum }\varepsilon_{ij}\left[{\left(\frac{R_{\min,\;ij}}{r_{ij}}\right)}^{12}-2{\left(\frac{R_{\min,\;ij}}{r_{ij}}\right)}^{6}\;\right] \end{array}$$

where *K*_*b*_, *K*_θ_, and K_χ_ are the bond, angle and torsional force constants; *b*, θ and χ are bond length, bond angle and dihedral angle (those with the 0-subscript are the equilibrium values); *n* is multiplicity and δ the phase of the torsional periodic function; *r*_*ij*_is the distance between atoms *i* and *j*; *q*_*i*_ and *q*_*j*_ are the partial charges of atoms *i* and *j*; ε is the effective dielectric constant; ε_*ij*_is the Lennard-Jones well depth and *R*_*min, ij*_ is the distance between atoms at Lennard-Jones minimum.

These terms may appear slightly different in different force-fields, and anharmonicity and cross-terms are generally added.

The parameters of the force field are obtained by fitting quantum mechanical or experimental values.

Examples of force field based scoring functions are GoldScore (Verdonk et al., 2003), AutoDock (Morris et al., 1998) (improved as a semiempirical version in AutoDock4, Huey et al., 2007), GBVI/WSA (Corbeil et al., 2012).

2. Empirical scoring functions:

These functions are the sum of various empirical energy terms such as van der Waals, electrostatic, hydrogen bond, desolvation, entropy, hydrophobicity, etc., which are weighted by coefficients optimized to reproduce binding affinity data of a training set by least squares fitting (Huang and Zou, 2010).

The LUDI (Böhm, 1994) scoring function was the first example of an empirical one. Other empirical scoring functions are GlideScore (Halgren et al., 2004; Friesner et al., 2006), ChemScore (Eldridge et al., 1997), PLANTS_CHEMPLP_ (Korb et al., 2009).

3. Knowledge-based scoring functions:

These methods assume that ligand-protein contacts statistically more explored are correlated with favorable interactions. Starting from a database of structures, the frequencies of ligand-protein atom pairs contacts are computed and converted into an energy component. When evaluating a pose, the aforementioned tabulated energy components are summed up for all ligand-protein atom pairs, giving the score of the pose.

DrugScore (Gohlke et al., 2000; Velec et al., 2005) and GOLD/ASP (Mooij and Verdonk, 2005) are examples of knowledge-based scoring functions.

Another strategy consists in the combination of multiple scoring functions leading to the so-called consensus scoring (Charifson et al., 1999).

In addition, new scoring functions have been developed: for example, based on machine learning technologies, interaction fingerprints and attempts with quantum mechanical scores (Yuriev et al., 2015).

#### Sampling

The first molecular docking algorithm was developed in the 1980s by Kuntz et al. (1982); the receptor was approximated by a series of spheres filling its surface clefts, and the ligand by another set of spheres defining its volume. A search was made to find the best steric overlap between binding site and receptor spheres, neglecting any kind of conformational movement.

**Figure 1:**
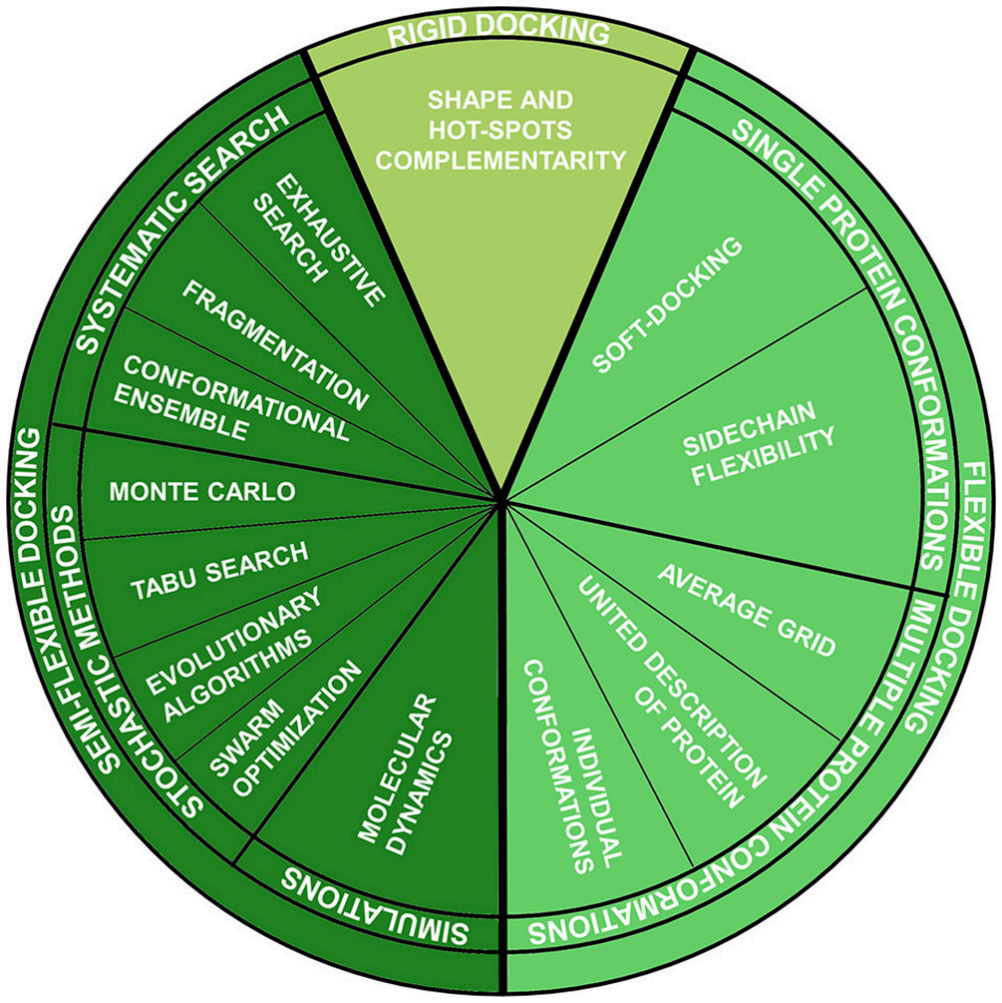
Molecular docking techniques organized according to ligand-protein flexibility and conformational searching engines.

This method belongs to the group of fully-rigid docking techniques, according to the classification which divides docking methods according to the degrees of flexibility of the molecules involved in the calculation Halperin et al., 2002 (Figure 1):

1. Rigid docking:

Both ligand and protein are considered rigid entities, and just the three translational and three rotational degrees of freedom are considered during sampling. This approximation is analogous to the “lock-key” binding model and is mainly used for protein-protein docking, where the number of conformational degrees of freedom is too high to be sampled. Generally, in these methods, the binding site and the ligand are approximated by “hot” points and the superposition of matching point is evaluated (Taylor et al., 2002).

2. Semi-flexible docking:

Just one of the molecules, the ligand, is flexible, while the protein is rigid. Thus, the conformational degrees of freedom of the ligand are sampled, in addition to the six translational plus rotational ones. These methods assume that a fixed conformation of a protein may correspond to the one able to recognize the ligands to be docked. This assumption, as already reported, is not always verified.

3. Flexible docking:

It is based on the concept that a protein is not a passive rigid entity during binding and considers both ligand and protein as flexible counterparts. Different methods have been introduced during the years, some rested on the induced fit binding model and others on conformational selection.

The great number of degrees of freedom introduced by flexible docking makes the potential energy surface to be a function of numerous coordinates. Consequently, the computational effort required to perform a docking calculation is augmented, but both sampling and scoring should be optimized to give a good balance between accuracy and speed. In fact, virtual screening campaign of millions of compounds depends on the velocity of docking calculations. For this reason, more and more improvements have been made in the development of the new algorithm, able to deeply search the phase space but not at the expense of velocity.

#### Semi-flexible docking

Numerous docking algorithms have been developed since the 1980s. Often it is difficult to classify clearly each docking software, because different algorithms may be integrated into a multi-phase approach. However, docking algorithms can be classified as follows (Kitchen et al., 2004; Huang and Zou, 2010):

1. Systematic search techniques:

In a systematic search, a set of discretized values is associated with each degree of freedom, and all the values of each coordinate are explored in a combinatorial way (Brooijmans and Kuntz, 2003). These methods are subdivided into:

- *Exhaustive search -* it is a systematic search in the strict sense since all the rotatable bonds of the ligands are examined in a systematic way. A number of constraints and termination criteria is generally established to limit the search space and to avoid a combinatorial explosion. The docking pipeline of the software Glide (Friesner et al., 2004; Halgren et al., 2004) involves a stage of the exhaustive search.
- *Fragmentation*- the first implementation of ligand flexibility into docking was introduced by DesJarlais et al. (1986), who proposed a method made of fragmentation of the ligand, rigid docking of the fragments into the binding site, and subsequent linking of the fragments. In this way, partial flexibility is implemented at the joints between the fragments. Other methods, defined as incremental construction, dock one fragment first and then attach incrementally the others. Examples of methods utilizing fragmentation are FlexX (Rarey et al., 1996) and Hammerhead (Welch et al., 1996).
- *Conformational Ensemble*- rigid docking algorithms can be easily enriched by a sort of flexibility if an ensemble of previously generated conformers of the ligand is docked to the target, in a sort of conformational selection fashion on the ligand counterpart. Examples are offered by FLOG (Miller et al., 1994), EUDOC (Pang et al., 2001), MS-DOCK (Sauton et al., 2008).

2. Stochastic methods:

Stochastic algorithms change randomly, instead of systematically, the values of the degrees of freedom of the system. The advantage of these techniques is the speed, so they could potentially find the optimal solution really fast. As a drawback, they do not ensure a full search of the conformational space, so the true solution may be missed. The lack of convergence is partially solved by increasing the number of iterations of the algorithm. The most famous stochastic algorithms are (Huang and Zou, 2010):

- *Monte Carlo (MC) methods*- Monte Carlo methods are based on the Metropolis Monte Carlo algorithm, which introduces an acceptance criterion in the evolution of the docking search. In particular, at every iteration of the algorithm, a random modification of the ligand degrees of freedom is performed. Then, if the energy score of the pose is improved, the change is accepted, otherwise, it is accepted according to the probability expressed in the following equation:
  $$\begin{array}{l} P\sim \text{exp}\left[\frac{-\left(E_{1}-E_{0}\right)}{k_{B}T}\right] \end{array}$$
  where *E*_1_ and *E*_0_ are the energy score before and after the modification, *k*_*B*_ the Boltzmann constant, and T the temperature of the system.

This is the original form of the Metropolis algorithm, but it is implemented in different variants within docking software. Some example are provided by the earlier versions of AutoDock (Goodsell and Olson, 1990; Morris et al., 1996), ICM (Abagyan et al., 1994), QXP (McMartin and Bohacek, 1997), MCDOCK (Liu and Wang, 1999), AutoDock Vina (Trott and Olson, 2010), ROSETTALIGAND (Meiler and Baker, 2006).

b. *Tabu search methods*- the aim of these algorithms is to prevent the exploration of already sampled zones of the conformational/positional space. Random modifications are performed on the degrees of freedom of the ligand at each iteration. The already sampled conformations are registered, and when a new pose is obtained, it is accepted only if not similar to any previously explored pose. PRO_LEADS (Baxter et al., 1998) and PSI-DOCK (Pei et al., 2006) are two examples of this category.
c. *Evolutionary Algorithms* (EA) - these algorithms are based on the idea of biological evolution, with the most famous Genetic Algorithms (GAs). The concept of the gene, chromosome, mutation, and crossover are borrowed from biology. In particular, the degrees of freedom are encoded into genes, and each conformation of the ligand is described by a chromosome (collection of genes), which is assigned a fitness score. Mutations and crossovers occur within a population of chromosomes, and chromosomes with higher fitness survive and replace the worst ones. The most famous examples are GOLD (Jones et al., 1995, 1997), AutoDock 3 & 4 (which implement a different version of GA, the Lamarckian GA) (Morris et al., 1998), PSI-DOCK (Pei et al., 2006), rDock (Ruiz-Carmona et al., 2014).
d. *Swarm optimization (SO) methods*- these methods take inspiration from swarm behavior. The sampling of the degrees of freedom of a ligand is guided by the information deposited by already sampling good poses. For example, PLANTS (Korb et al., 2006) adopts an ACO (Ant Colony Optimization) algorithm, which mimics the behavior of ants, who communicate the easiest way to reach a source of food through the deposition of pheromone. Here, each degree of freedom is associated with a pheromone. Virtual ants choose conformations considering the values of pheromones, and successful ants contribute to pheromone deposition.

Other examples of SOs are SODOCK (Chen et al., 2007), pso@autodock (Namasivayam and Günther, 2007), PSOVina (Ng et al., 2015).

3. Simulation methods:

The most famous example of this category is Molecular Dynamics, a method that describes the time evolution of a system. A wider explanation will be given in section Molecular Dynamics.

Energy minimization methods can be inserted in this category, but generally, they are not used as stand-alone search engines (Kitchen et al., 2004). Energy minimization is a local optimization technique, used to bring the system to the closest minimum on the potential energy surface.

#### Flexible docking

Some attempts have been made to introduce protein flexibility into docking calculations. These methods take advantage of different degrees of approximation and can be divided into approaches that consider single protein or multiple protein conformations (Alonso et al., 2006).

1. Single Protein Conformation:

a. Soft docking:

This method, firstly described by Jiang and Kim (1991), consists of an implicit and rough treatment of protein flexibility. The van der Waals repulsion term employed in force field scoring functions is reduced, allowing small clashes that permit a closer ligand-protein packing. In this way, a sort of induced-fit is simulated. As a drawback, this approach approximates just feeble protein movements and could implicate unreal poses (Apostolakis et al., 1998; Vieth et al., 1999).

b. Sidechain flexibility:

This strategy introduces alternative conformations for some protein side chains (Leach, 1994). This is generally done exploiting databases of rotamer libraries. Some docking methods, such as GOLD, sample some degrees of freedom within their own search engine. Obviously, considering side chain flexibility, huge conformational variations of the protein are neglected by these methods.

2. Multiple Protein Conformations:

Multiple experimental structures may be available for the same target. Moreover, an ensemble of protein conformations can be obtained via computational techniques, such as Monte Carlo or Molecular Dynamics simulations. The idea of multiple protein conformations docking is to take into account all the diverse structures, following different possible strategies:

a. Average grid:

The structures of the ensemble are used to construct a single average-grid, which can be either a simple or weighted average combination of them (Knegtel et al., 1997).

b. United description of the protein:

In this case, the structures do not collapse into an average grid but are used to construct the best performing “chimera” protein. For example, FlexE (Rarey et al., 1996) extracts the structurally conserved portions from the structures of the ensemble and uses them to construct an average rigid structure. This portion is fused to the flexible parts of the ensemble in a combinatorial fashion, giving a pool of “chimeras” that are used for docking.

c. Individual conformations:

The structures of the ensemble are considered as conformations that can possibly be bound by the ligand, so various docking runs are performed, evaluating the ligands of interest on all the target conformations (Huang and Zou, 2007). Moreover, a preliminary benchmark assessing the performance of different target structures in a cross-docking experiment may be employed to filter the ensemble of structures (Salmaso et al., 2016, 2018).

Among the drugs approved by the Food and Drug Administration, few examples of successful applications of CADD are available (Talele et al., 2010). Among them, the renin-inhibitor Aliskiren was developed by means of a combination of molecular modeling and crystallographic structure analysis (Wood et al., 2003). However, the binding of non-peptidomimetic ligands to renin has shown huge structural rearrangement of the protein (Teague, 2003), addressing the problem of considering protein flexibility in drug design campaigns. Recently, a comparative study evaluating the performance of ensemble docking and individual crystal structure docking has been proposed for renin (Strecker and Meyer, 2018). An ensemble of 4 crystal structures outperformed the mean results of individual crystal structures in terms of binding mode prediction and screening utility. The ensemble gave worse results than the best performing crystal structure, which though is not known a priori. Not as good results were obtained through a Molecular Dynamics ensemble when compared to crystallographic structures, as confirmed in other cases reported in the literature (Osguthorpe et al., 2012; Ganser et al., 2018). However, Molecular Dynamics has proven to be effective as a tool to explore molecular conformations and as a docking method itself, as reported in the following paragraphs.

### Molecular dynamics

Molecular dynamics (MD) is a computational technique which simulates the dynamic behavior of molecular systems as a function of time, treating all the entities in the simulation box (ligand, protein, as long as waters if explicit) as flexible (Salmaso, 2018).

It was developed to simulate simple systems, with the first application to study collisions among hard spheres, in 1957 (Alder and Wainwright, 1957). The first MD simulation of a biomolecule was accomplished in 1977 by McCammon et al. (McCammon et al., 1977); it was a 9.2 ps simulation of a 58-residues Bovine Pancreatic Trypsin Inhibitor (BPTI), performed in vacuum with a crude molecular mechanics potential.

**Figure 2:**
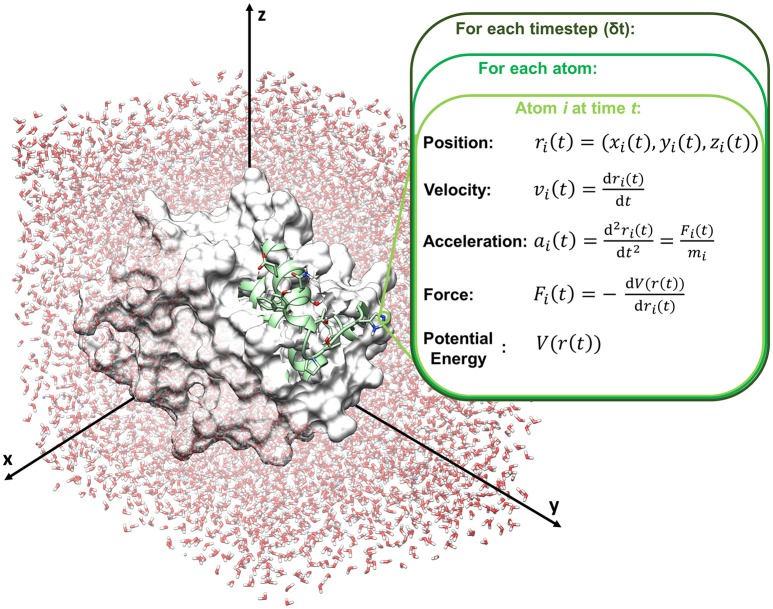
Schematic representation of a molecular dynamics cycle.

Molecular dynamics compute the movements of atoms along time by the integration of Newton's equations of motions (classical mechanics), reported in the following equation (Leach, 2001; Adcock and McCammon, 2006).

$$\begin{array}{l} \frac{d^{2}r_{i}\left(t\right)}{dt^{2}}=\;\frac{F_{i}\left(t\right)}{m_{i}} \end{array}$$

with *F*_*i*_*(t)* force exerted on atom *i* at time *t, r*_*i*_*(t)* vector position of the atom *i* at time *t, m*_*i*_ mass of the atom (Figure 2).

In particular, time is partitioned into time steps (δ*t*), which are used to propagate the system forward in time. Several integration algorithms are available, which derive Newton's equations by a discrete-time numerical approximation. The velocity-Verlet integrator is reported in the following equations as an example to compute position and velocity of an atom *i* at the time step *t*+δ*t*, starting from step *t*.

$$\begin{array}{l} r_{i}\left(t+\delta t\right)=\;r_{i}\left(t\right)+\;v_{i}\left(t\right)\delta t+\;\frac{1}{2}a_{i}\left(t\right)\delta t^{2}\\ v_{i}\left(t+\delta t\right)=\;v_{i}\left(t\right)+\;\frac{1}{2}\;\left[a_{i}\left(t\right)+\;a_{i}\left(t+\delta t\right)\right]\delta t \end{array}$$

where *r*_*i*_*(t), v*_*i*_*(t)* and *a*_*i*_*(t)* are respectively position, velocity and acceleration of atom *i* at time *t*, and *r*_*i*_*(t*+δ*t), v*_*i*_*(t*+δ*t)* and *a*_*i*_*(t*+δ*t)* are respectively position, velocity and acceleration of atom *i* at time *t*+δ*t*.

Acceleration is calculated from the forces acting on atom *i* according to Newton's second law, and forces are computed from the force field, according to the following equation:

$$a_{i}\left(t\right)=\;\frac{d^{2}r_{i}(t)}{dt^{2}}=\;\frac{F_{i}(t)}{m_{i}}=\;-\;\frac{dV\left(r\left(t\right)\right)}{m_{i}dr_{i}(t)}$$

where *V(r(t))* is the potential energy function retrieved by the force field (see Box 1).

The most used force fields in molecular dynamics are CHARMM (MacKerell et al., 1998), AMBER (Cornell et al., 1995), OPLS (Jorgensen and Tirado-Rives, 1988) and GROMOS (Oostenbrink et al., 2004).

#### Molecular dynamics and exploration of the phase space

MD trajectories can be used as sampling engines; in fact, they produce protein conformations usable for Multiple Protein Conformations docking applications. In particular, McCammon et al. developed the so-called Relaxed-Complex Scheme (RCS), consisting in docking mini-libraries of compounds with AutoDock (Morris et al., 1998) against a large ensemble of snapshots derived from unliganded protein MD trajectories (Lin et al., 2002, 2003; Amaro et al., 2008). This approach is based on the conformational selection binding model, disregarding any influence of the ligand on the receptor. The application of the RCS to the UDP-galactose 4′-epimerase (*Tb*GalE), for example, led to the identification of 14 low-micromolar inhibitors (Durrant et al., 2010). Another computational pipeline integrating MD simulations and virtual screening has proved to be effective: the coupling of MD, clustering, and choice of the target structure through fingerprints for ligand and proteins (MD-FLAP) improved VS performance (Spyrakis et al., 2015).

MD has further applications as a docking-coupled technique (Alonso et al., 2006) more anchored to the induced-fit model, as it can be used to assess stability (Sabbadin et al., 2014; Yu et al., 2018), to refine and to rescore docking poses (Rastelli et al., 2009).

The relevance of MD simulations as source of target conformational profusion can be exploited to retrieve insights into cryptic pockets or allosteric binding sites (Durrant and McCammon, 2011), as reported by Schame et al., who identified an alternative binding site, named “trench,” close to the active site of the HIV-1 integrase (Schames et al., 2004). Moreover, simulations in the explicit solvent may give information on water molecules, that can be classified as “cold” or stable and “hot” or unstable (for a recent and comprehensive overview on the role of water in SBDD; see Spyrakis et al., 2017). In particular, MD may enable to individuate relevant water molecules, according to their order (Li and Lazaridis, 2003) and stationarity (Cuzzolin et al., 2018), and to estimate their contribution in modulating ligand binding (Bortolato et al., 2013; Betz et al., 2016).

All the aforementioned applications of MD are used as a complement to classic molecular docking techniques. however, the simulation of the complete binding process of a ligand, from the unbound state in bulk solvent to the bound state, be considered a fully-flexible docking in explicit solvent. The possibility to investigate the whole binding process could give insights into metastable states reached by the ligand during the simulation, alternative binding sites, the role of water during binding and conformational rearrangements preceding, concurrent or consecutive to binding.

However, the observation of a binding event during a classical MD simulation is very rare, raising the timescale problem. The timestep in molecular dynamics has to be compatible with the fastest motion in the system; in particular, a timestep of 1–2 fs, corresponding to bond vibrations, has to be used. Thus, a high number of MD steps is required to simulate slow processes, such as large domain motions and binding (μs-ms) (Henzler-Wildman and Kern, 2007), making the computational effort really hard. In particular, slow timescale are linked to processes that require the overcoming of a high energy barrier (Henzler-Wildman and Kern, 2007), corresponding to low populated states in the conformational energy landscape; in this case the simulated system gets trapped in a local minimum, making classical MD inadequate to explore largely the conformational space.

#### Advances in classical MD simulations

In 1998 Duan and Kollman performed the first 1μs simulation of a protein in explicit solvent, observing the folding of a 36-residue villin headpiece subdomain from a fully unfolded state. This simulation was two orders of magnitude longer than a state-of-the-art simulation of that period, and it was made possible by advances in massively parallel supercomputers and efficient parallelized codes, but still required 2 months of CPU (Central Processing Units) time (Duan and Kollman, 1998).

Specialized informatic infrastructures have also been designed specifically for MD calculations; for example, a supercomputer named Anton was conceived as a “computational microscope” and was developed with the idea to reach previously inaccessible simulation timescales within a reasonable computation time (Shaw et al., 2008). This machine allowed Shaw et al. to characterize the folding of FiP35 WW domain from a fully extended state in a 100 μs simulation and, in addition, to reach the millisecond timescale in a single simulation of BPTI in the folded-state (Shaw et al., 2010), followed recently by ubiquitin (Lindorff-Larsen et al., 2016). Moreover, with unbiased simulations in the order of ten microseconds, Shaw's group could simulate the complete binding process of beta blockers and agonists to the β_2_-adrenergic receptor (Dror et al., 2011) and kinase inhibitors to Src kinase (Shan et al., 2011).

As a drawback, the utilization of supercomputer is an expense that not many research groups can afford. Fortunately, the recent years have been characterized by the development of code able to exploit the speed of GPUs (Graphics Processing Units), which has given access to tera-scale performances with the use of a common workstation, and a consequent relatively low cost (Van Meel et al., 2008; Friedrichs et al., 2009; Harvey et al., 2009; Nobile et al., 2017). The architecture of a GPU is meant to parallelize a computation over thousands of cores, with all cores executing the same instructions on different data (“Same Instruction Multiple Data,” SIM) (Nobile et al., 2017). For this reason, together with few preliminary applications in the field of molecular docking (Korb et al., 2011; Khar et al., 2013), GPUs have been mainly exploited for MD simulations, which can be parallelized at the level of atoms. In fact, nowadays, simulations of hundreds of nanoseconds are easily performed, and reaching the microsecond timescale is an affordable issue on a GPU-equipped workstation (Harvey and De Fabritiis, 2012). In addition, cloud computing has been emerging nowadays, not just through the use of web-servers intended to make molecular modeling accessible to a community of non-developers users, but also with the provision of computation power scalable and on-demand (Ebejer et al., 2013). As an example, AceCloud is an on-demand service for MD simulations, which is accessed through an extension of the ACEMD MD code (Harvey and De Fabritiis, 2015).

Moreover, a paradigm shift seems to have been spreading, that is the possibility to simulate long processes using numerous trajectories shorter than the process itself instead of a single long trajectory. This idea has been exploited by the folding@home project, a worldwide distributed computing environment benefitting from the computers of private citizens, when not in use (Shirts and Pande, 2000). Since during a classical MD simulation, the system is stuck in a minimum, waiting for the fortunate event that triggers the overcoming of an energy barrier, the simulation of many trajectories in parallel would increase the probability to meet the lucky event. Thus, numerous simulations are started from the same initial condition and run in parallel on different computers, and when one escapes from the energy minimum, all the simulations are stopped and started from the new productive configuration (Pande et al., 2003).

The new paradigm has found its best application in the use of Markov State Models (MSMs) and adaptive sampling. In fact, MSMs are based on an ensemble view of the dynamics, from which statistical properties, such as the probability to occupy a state and the probability to jump from one state to another, are computed. The construction of a Markov model is made of the discretization and projection of a trajectory into microstates, and of a transition probability matrix T(τ) computation at a given time, the lag-time τ, chosen in a way that the transition is memory-less (Markovian). Each element T_*ij*_(τ) of the transition matrix represents the conditional probability to find the system in state *j* at time *t*+ τ while being in state *i* at time *t*. The transition matrix approximates the dynamic of the system and enables to extrapolate the free energy from the equilibrium probability distribution of the system and the timescale of the slowest processes, even if they are not directly explored. In a qualitative fashion, the MSM may individuate diverse metastable states and construct multi-states models of the processes (Prinz et al., 2011). As an example, an MSM was constructed on an aggregate of nearly 500 100 ns-trajectories describing benzamidine-trypsin binding (with 37% productive trajectories); this enabled to characterize the binding process individuating three transition states, and to estimate binding free energy with 1 kcal/mol difference from the experimental one (while a higher deviation from experiment was associated with the extrapolated k_on_ and k_off_) (Buch et al., 2011). Moreover, the computation of MSM on the collected data can give a feedback about undersampled zones of the phase space, suggesting where to focus further simulation, adapting the sampling (adaptive sampling methods) and increasing the efficiency of simulations (Bowman et al., 2010; Doerr and De Fabritiis, 2014). Currently, the major difficulties of this technique are related to the trajectory partition into discrete states, the choice of the lagtime and sufficient sampling to guarantee statistical significance (Pande et al., 2010).

Several alternative techniques have been developed during the years to overcome the time limitation imposed by classical MD simulations. A first example consists of the Coarse-Grained MD simulations, in which groups of atoms are condensed into spheres, reducing the degrees of freedom of the system (Kmiecik et al., 2016). This simplifies the conformational landscape of the system, but, as a drawback, the information on the all-atom simulations, that are precious for drug-discovery aim, are lost.

Additional strategies consist of enhanced sampling techniques that apply a bias to molecular dynamics simulations to increase the accessible timescale, enabling the simulation of slow processes like binding, unbinding and folding processes in a reduced amount of time.

#### Enhanced sampling techniques

These methods add a bias force/potential to the system to increase the rate of escape from local minima, entailing an acceleration of conformational sampling. They have been conceived primarily to study either folding or binding or unbinding processes, sharing the underlying idea of enhancement of sampling and overcoming high energy barriers.

**Figure 3:**
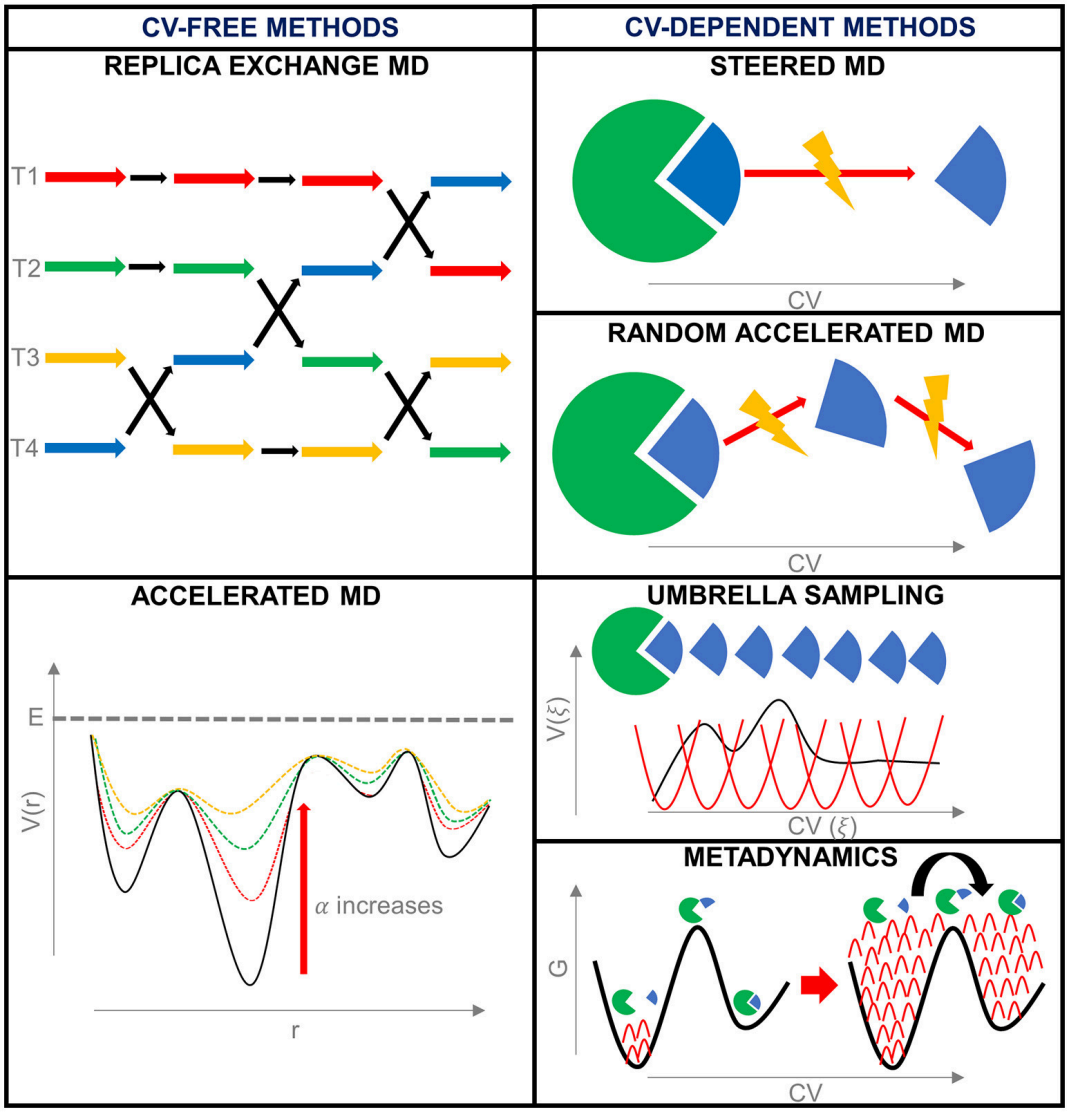
Summary of the enhanced sampling techniques described during this work.

Enhanced sampling techniques can be divided into methods that make use of collective variables to introduce the bias and methods that do not (De Vivo et al., 2016) (Figure 3).

The employment of a collective variable (CV) is based on the idea that a complex system can be decomposed into one or a combination of reaction coordinates describing the process of interest. These coordinates are named as collective variables since it is assumed they can summarize the behavior of the entire system. After a careful choice of the CVs, the bias is added on these coordinates during the simulation enhancing sampling along the CVs. The phase space is reduced to the space of the collective variables, since the conformational space is projected to the selected CVs, with a consequent dimensional reduction of the free energy surface.

In the following paragraphs, few representative enhanced sampling techniques are reported as an example, focusing on their application in binding and unbinding and going toward a fully dynamic docking (De Vivo and Cavalli, 2017).

##### Collective variables-free methods

*Replica Exchange Molecular Dynamics (REMD)* This method adopts an increase in temperature to accelerate the conformational sampling. The first formulation of Replica Exchange MD (Sugita and Okamoto, 1999), also known as Parallel Tempering (PT), consists of the parallel simulation of a number of independent and simultaneous replicas of the same system, starting from the same configuration, but at different temperatures. At regular time intervals, two replicas characterized by neighbor temperatures are switched, or, in other terms, their temperatures are exchanged, with a probability determined by the energy (*E*) and temperature (*T*) of the system. In particular, the transition probability between simulations at temperature *T*_1_ and *T*_2_ is determined by the Metropolis criterion:

$$P\left(T_{1}\;➔\;T_{2}\right)=\;\{\begin{array}{c} \;1\;for\;\left[\beta_{2}-\beta_{1}\right]\left(E_{1}-E_{2}\right)\;\leq 0\\ e^{-\left[\beta_{2}-\beta_{1}\right]\left(E_{1}-E_{2}\right)}\;for\;\left[\beta_{2}-\beta_{1}\right]\left(E_{1}-E_{2}\right)>0 \end{array}$$

where β = *1/k*_*B*_*T* (with *k*_*B*_ the Boltzmann constant).

Temperatures are updated by rescaling the velocities of the parent simulations (*v*_1_ and *v*_2_ to *v*_1_′ and *v*_2_′) according to the following equation:

$$\left\{ \begin{array}{l} v_{1}^{\prime }=\;\sqrt{\frac{T_{2}}{T_{1}}v_{1}}\\ v_{2}^{\prime }=\;\sqrt{\frac{T_{1}}{T_{2}}v_{2}} \end{array}\right.$$

The choice of the panel of temperatures is critical, and various strategies have been proposed to guide the selection (Patriksson and van der Spoel, 2008).

Further development of REMD has been introduced, such as the Hamiltonian Replica Exchange (H-REMD), where Hamiltonians are exchanged among replicas instead of temperatures (Fukunishi et al., 2002), and Replica Exchange with Solute Tempering, where a different treatment of the central group and the solvent buffer is performed (Liu et al., 2005). HREMD has been recently combined to conventional MD simulations using multi-ensemble Markov models (MEMMs) (Wu et al., 2016) to investigate the multistate kinetics of Mdm2 and its inhibitor peptide PMI (Paul et al., 2017). An ensemble of 500 μs unbiased MD simulations conducted from different initial states, especially dissociated, were combined to HREMD simulations (6 simulations of 1 μs and with 14 replicas) to enhance sampling of rare dissociation events; the results were analyzed through the TRAMMBAR estimator, leading to the prediction of a residence time beyond the second timescale, despite a sub-millisecond simulation time. Moreover, the trajectories were furtherly analyzed to investigate the binding mechanism and binding-induced folding of PMI (Paul et al., 2018). It appeared that a multitude of parallel pathways is possible and that binding and folding are coupled, while not temporarily ordered and separated.

*Accelerated Molecular Dynamics (AMD)* Accelerated MD (aMD) facilitates the egress from a low energy basin by adding a bias potential function (Δ*V(r)*) when the system is entrapped in an energy minimum. In particular, when the potential energy (*V(r)*) is lower than a certain cut-off (*E*), the bias is added giving a modified potential (V^*^(r) = *V(r)*+ Δ*V(r)*); otherwise the simulation continues in the true-unbiased potential (V^*^(r) = *V(r)*).

The bias function is reported in the following equation:

$$\begin{array}{l} \Delta V\left(r\right)=\;\frac{{\left(E-V\left(r\right)\right)}^{2}}{\alpha +\left(E-V\left(r\right)\right)} \end{array}$$

where *E* is the potential energy cut-off and α is a tuning parameter determining the depth of the modified potential energy basin.

*E* has to be at least greater than *V*_*min*_ (the minimum potential energy, close to the starting configuration), while α = *E*- *V*_*min*_ will allow maintaining the underlying shape of the landscape (Hamelberg et al., 2004).

As an example, aMD showed qualitatively similar results to classical MD with fewer computational effort in the simulation of tiotropium-M_3_ Muscarinic Acetylcholine Receptor binding: tiotropium was observed to recognize the extracellular vestibule of the receptor, as in a previously reported long (16 μs) classical MD simulation (Kruse et al., 2012), by accelerating the process of about one order of magnitude (three aMD replicas of 200 ns, 500 ns, and 1 μs) (Kappel et al., 2015).

##### Collective variables-dependent methods

*Steered Molecular Dynamics (SMD)* Taking inspiration from atomic force microscopy experiments, in Steered MD (SMD) an external force is applied to a ligand to drive it out of the target binding site (Isralewitz et al., 1997, 2001; Izrailev et al., 1997). Other possibilities involve the application of forces on different CVs, such as nonlinear coordinates that can help to explore the conformational rearrangement of protein domains (Izrailev et al., 1999).

SMD gives insights into the ligand-target unbinding mechanism, which can be investigated through the dynamical evolution of the ligand-target pattern of interactions, as reported for a series of Cyclin-Dependent Kinase 5 (CDK5) inhibitors (Patel et al., 2014). In the same work, the second application of SMD in drug discovery is highlighted: since the bias force added during an SMD simulation is assumed to be related to the binding strength, the binding force profile can be used to discriminate binders from non-binders.

SMD relies on an a priori definition of the applied force direction, which can be fixed (for example a simple straight line) or can change during the simulation. The choice of the direction is not trivial, because a ligand may bump into obstructions during its way out of the protein, but a method evaluating the minimal steric hindrance has been reported (Vuong et al., 2015). Moreover, integration with the targeted molecular dynamics (TMD) are reported: in TMD a bias force is applied to conduct the system from an initial to a desired final configuration (Schlitter et al., 1993), leading to the individuation of a path that can be used as set of directions for an SMD simulation (Isralewitz et al., 2001).

*Random Acceleration Molecular Dynamics (RAMD)* Random Acceleration MD (RAMD), also defined Random Expulsion MD, is an extension of SMD, and, like this, was developed to study the egress of a ligand from its target binding site. It consists of the application of an artificial randomly-directed force on a ligand to accelerate its unbinding. In this way, in comparison with SMD, RAMD avoids the preliminary choice of the force direction; consequently, if some obstructions are found during the exit pathway, the escape direction is switched.

In particular, the direction of the force is chosen stochastically and maintained for a number of MD steps. If during this time interval the average velocity of the ligand is lower than a specified cut-off (or, in other terms, if the distance covered by the ligand is lower than a cut-off distance, *r*_*min*_), meaning that probably a rigid obstruction has been met, a new force direction is assigned to allow the ligand to search for alternative exit pathways (Lüdemann et al., 2000).

As SMD, RAMD is predominantly used to simulate ligand unbinding from a molecular target. The egress of carazolol from β_2_ Adrenergic Receptor was for example described thanks to an ensemble of RAMD simulations (100 simulation, with a variable length of maximum 1 ns): the extracellular surface opening of the receptor was individuated as the predominant exit root, entailing the rupture of a salt bridge linking extracellular loop 2 to transmembrane helix 7 (Wang and Duan, 2009).

*Umbrella Sampling (US)* Umbrella Sampling (US) (Torrie and Valleau, 1977) consists of restraining the system along one or a combination of CVs. Commonly, the range of interest of the CV is divided into windows, each characterized by a reference value of the CV (ξ_*ref*_). The bias potential enhances sampling in each window by forcing the system to stay close to the respective CV reference value. The bias is a function of the reaction coordinate, and can have different shapes, but generally consists of a simple harmonic, as in the following equation:

$$\begin{array}{l} V\left(\xi \right)=\;\frac{k}{2}\;{\left(\xi -\xi_{ref}\right)}^{2} \end{array}$$

Where *k* is the strength of the potential and ξ is the value of the CV.

The strength of the bias has to be high enough to let energy barriers crossing, but sufficiently low to enable the overlapping of system distributions of different windows, as required for post-processing analysis.

The aim of US is to force sampling in each window to collect sufficient statistics along with the whole reaction coordinate. Then the distribution of the system and consequently the free energy is calculated along the CV (Kästner, 2011). Different post-processing methods can be used to perform combination and analysis of the data coming from the different US windows; the most famous is umbrella integration (Kästner and Thiel, 2005), the weighted histogram analysis method (WHAM) (Kumar et al., 1992), and the more recent Dynamic Weighted Histogram Analysis (DHAM) (Rosta and Hummer, 2015), which can be used also to derive kinetic parameters.

Integrations of US with other enhanced sampling techniques are reported in the literature, such as the replica-exchange umbrella sampling method (REUS), where an umbrella potential is exchanged among replicas (Sugita et al., 2000; Kokubo et al., 2011). This technique was applied to the prediction of ligand-protein binding structures, starting from unbound initial states and employing as CV ξ the distance between the centers of mass of the ligand and of the backbone of two selected residues. This technique resulted to be effective in the prediction of the binding mode of a couple of ligands on p38 and JNK3 kinases (RMSD minor than 1.7 Å), and outperformed a cross-docking experiment, highlighting the importance of considering protein flexibility to accurately predict the coordinates of a complex (Kokubo et al., 2013).

*Metadynamics* Metadynamics (Laio and Parrinello, 2002) introduces a bias potential to the Hamiltonian of the system in the form of a Gaussian-shaped function of one or more CVs. In this case, the bias does not restrain or constrain the system, neither force the system along with a preferred direction in the CV space. The bias is used to keep the memory of the already explored zones of the phase space, and to discourage the system to visit them again (Laio and Gervasio, 2008).

At time t, the bias potential (*V*_*G*_*(S,t)*) is reported in the following equation:

$$\begin{array}{l} V_{G}\left(S,t\right)=\;\int_{0}^{t}dt^{{\;}^{\prime }}\omega \exp\left(-\overset{d}{\underset{i=1}{\sum }}\frac{{\left(S_{i}\left(R\right)-\;S_{i}\left(R\left(t^{{\;}^{\prime }}\right)\right)\right)}^{2}\;}{2\sigma_{i}^{2}}\right) \end{array}$$

where *S(R)* = (*S*_1_*(R)*,…,*S*_*d*_*(R)*) is a set of *d* CVs (which are functions of the coordinates *R* of the system), *S*_*i*_*(R(t))* is the value of the *i*th CV at time *t*, σ_*i*_ is the Gaussian width for the *i*th CV, and ω is the energy rate, given by:

$$\begin{array}{l} \omega =\;\frac{W}{\tau_{G}} \end{array}$$

with *W* the Gaussian height and τ_G_ the deposition rate.

Thus, the bias is “history-dependent,” because it is the sum of the Gaussians that have already been deposited in the CV space during the time.

The free energy landscape is explored, starting from the bottom of a well, by a random walk; bias-Gaussians are deposited in the CV space with a given frequency, and at each iteration, the bias is given by the sum of the already deposited Gaussians. As time goes by, the system, instead of being trapped in the bottom of a well, is pushed out by the hill of deposited Gaussians and enters a new minimum. The process continues until all the minima are compensated by the bias potential (Barducci et al., 2011).

Metadynamics in this way enables to enhance sampling and to reconstruct the free energy surface; this can be used to explore binding/unbinding processes (Gervasio et al., 2005), and, with the application of funnel metadynamics (Limongelli et al., 2013), to the estimation of binding free energy.

Unfortunately, it may occur that the free energy surface is overfilled, but this has been partially solved by well-tempered metadynamics, in which the height of the added Gaussian is rescaled by the already deposited bias (Barducci et al., 2008). Another issue with metadynamics is the choice of the CVs, which should describe the slowest motions of the system and the initial-final-relevant intermediates. Moreover, a small number of CVs has to be used, and a good strategy is a combination with other techniques able to enhanced sampling along a great number of transverse coordinates (Barducci et al., 2011), such as with parallel tempering (Bussi et al., 2006). Using a well-tempered multiple-walker funnel-restrained metadynamics, the binding pathway of several ligands to 5 G-protein-coupled receptors (including X-ray crystal structures and homology models) has been recently explored, resulting in the prediction of binding free energies with a root-mean-square error minor than 1 kcal mol^−1^ (Saleh et al., 2017).

#### Supervised molecular dynamics

In the last years, a new method, called Supervised Molecular Dynamics (SuMD), has been introduced to accelerate the binding process (Sabbadin and Moro, 2014; Cuzzolin et al., 2016). SuMD is distinguished from enhanced sampling simulations since it does not affect the energy profile of the system.

**Figure 4:**
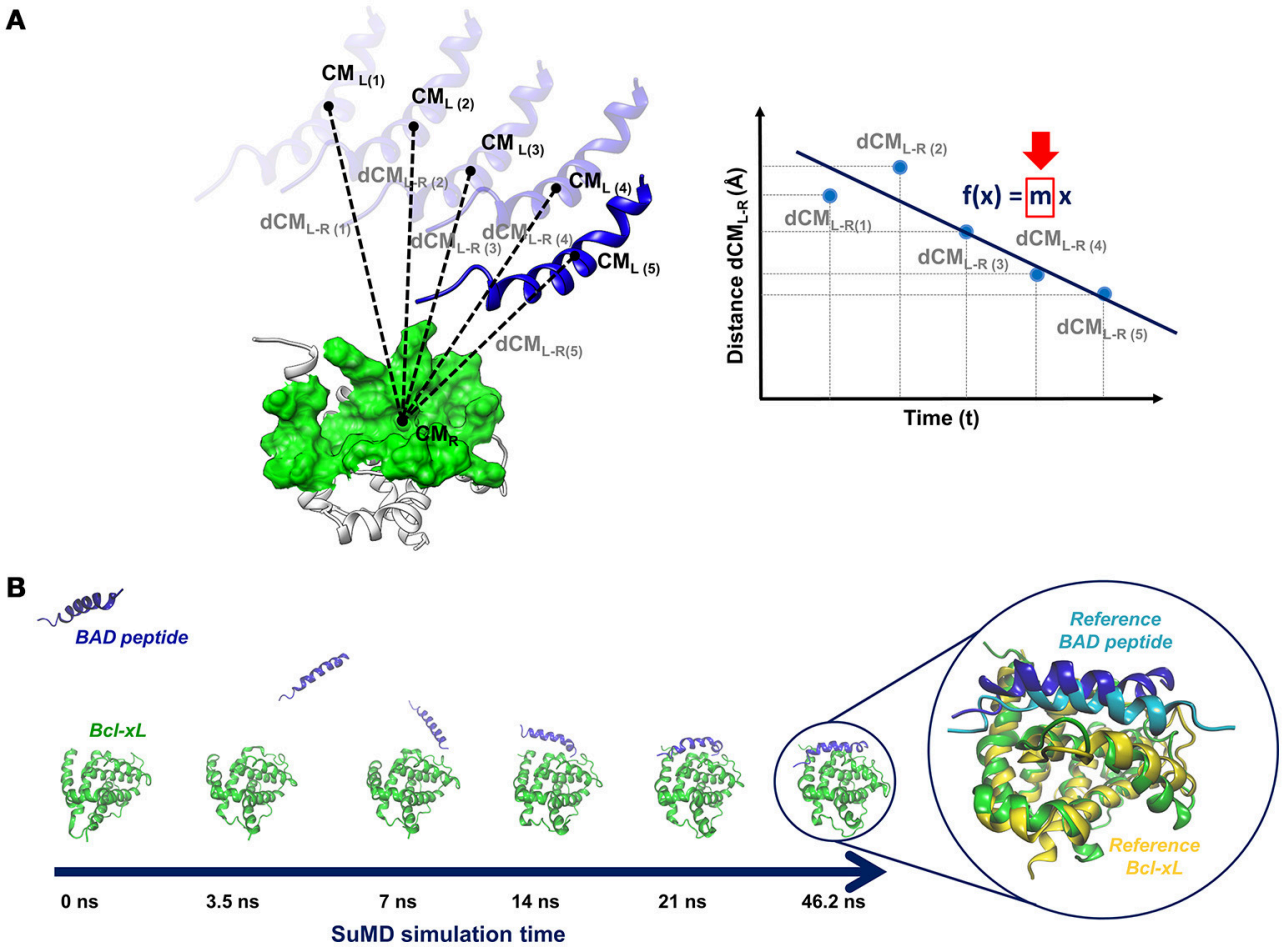
**(A)** Sketch of a pepSuMD step: the distance between the centers of mass of the ligand (peptide) and the target is computed at regular time intervals during the SuMD step. The distance values are fitted by a line, whose slope (m) determines if the current SuMD step (m > 0) or a new one (m < 0) has to be simulated. **(B)** Representation of the binding pathway bringing BAD peptide to the Bcl-X_L_ binding site, occurring in 46.2 ns. The superposition of the final pepSuMD state with the experimental structure (PDB ID: 1G5J, Petros et al., 2000) is reported on the right.

A SuMD simulation consists of a series of small MD windows (hundreds of picoseconds), called SuMD steps, where step *n*+*1* is run after the evaluation of step *n* in terms of ligand-target approaching. During each SuMD step, the distance between the centers of mass of the ligand and of the target binding site (few selected residues) is computed; distance values are collected at regular intervals during the simulation and are fitted by a line (Figure 4A). If the slope of the line is negative, it means that the ligand is approaching the binding site, the SuMD step (step *n*) is considered productive, and a new step (step *n*+*1*) is started from the last coordinates and velocities of the current step. Otherwise, if the slope is positive, it means that the SuMD step is unproductive, thus the current SuMD step simulation is deleted and restarted from its initial coordinates (starting configuration of step *n*). The simulation is concluded after that the distance between the centers of mass of ligand and target fall under a certain cut-off. Finally, the consecutive SuMD steps are merged together providing the SuMD trajectory.

In this way, SuMD enables to observe a binding event in a reduced timescale, in the orders of tens to hundreds of nanoseconds, without the introduction of any energetic bias. Indeed, SuMD simply focuses sampling by the introduction of a tabu-like algorithm which favors the progress of a simulation toward productive events and avoids wasting simulation time in uninteresting portions of the search space.

Certainly, a single SuMD trajectory is not sufficient to explain the complex binding process, and the retrieval of thermodynamic quantities from a single simulation must be avoided. Nevertheless, a SuMD trajectory depicts one of the possible binding pathways leading a ligand to reach the target, so it can be useful to propose a mechanistic hypothesis.

The technique was first applied to Adenosine Receptors, where it facilitated the characterization of the binding pathways of several ligands toward the receptor, with the exploration of metabinding sites (Sabbadin and Moro, 2014; Sabbadin et al., 2015). In this context, SuMD can be useful in the interpretation of allosteric interactions (Deganutti et al., 2015) and has proved to be supportive to the identification of fragment-like positive allosteric modulators (Deganutti and Moro, 2017). In fact, SuMD turned out to be effective in simulating fragment compounds, as shown by the accurate prediction of the binding mode of a catechol fragment to human peroxiredoxin 5 (PRDX5), reaching a minimum RMSD of 0.7 Å from the crystallographic pose.

The applicability spectrum of SuMD has been furtherly enlarged, till the development of pepSuMD, a revised version of the technique able to simulate the binding pathway of a peptide ligand toward its protein binding site (Salmaso et al., 2017). The recognition process of the BAD peptide to Bcl-X_L_ protein (Figure 4B) and of the p53 peptide to MDM2 has been recently reported, with the achievement of an RMSD less than 5 Å from the experimental conformation in tens of nanoseconds in both cases (46.2 and 23.40 ns, respectively). During the BAD/Bcl-X_L_ simulation, the C-terminal helix explored different conformations, meaning that peptide and protein conformational rearrangements can be observed during a SuMD simulation when occurring in the same time scale of the SuMD-accelerated binding.

## Conclusions and perspectives

In this review, an excursus over some relevant computational techniques in drug discovery has been performed, highlighting how protein flexibility has been introduced into the simulations during the years. Starting from simple rigid docking strategies justified by the lock-key model, it was soon necessary to consider conformational degrees of freedom of ligands during docking. Experimental data proving the existence of different conformations of protein structures has made the molecular models to face the problem of interpreting and simulating conformational transitions of macromolecules.

From rough attempts to include protein flexibility during classical molecular docking, the development of hardware technologies and of novel MD computational techniques has been allowing more and more to simulate huge conformational movements. The possibility to simulate contemporary folding and binding phenomena can be exploited to answer the long-standing debate about “induced-fit” and “conformational selection” binding models, by giving a mechanistic interpretation of binding pathways.

Moreover, some of the enhanced sampling techniques are no more an exclusive methodological exercise, but has become within reach of many research groups, whit a consequent real applicability in drug discovery.

## Author contributions

VS and SM devised the organization, the main conceptual ideas, proof outline and wrote the review. The content of the present work has been largely taken from the PhD thesis entitled Exploring protein flexibility during docking to investigate ligand-target recognition written by VS under the supervision of SM.

### Conflict of interest statement

The authors declare that the research was conducted in the absence of any commercial or financial relationships that could be construed as a potential conflict of interest.
